# Validation of a predictive model for speech discrimination after cochlear impIant provision

**DOI:** 10.1007/s00106-023-01285-y

**Published:** 2023-05-04

**Authors:** Ulrich Hoppe, Anne Hast, Thomas Hocke

**Affiliations:** 1 Audiologische Abteilung, Hals-Nasen-Ohrenklinik, Kopf- und Halschirurgie, Universitätsklinikum Erlangen, Waldstr. 1, 91054 Erlangen, Germany; 2 Cochlear Deutschland GmbH & Co. KG, Mailänder Str. 4a, 30539 Hannover, Germany

**Keywords:** Speech discrimination tests, Speech audiometry, Hearing tests, Hearing loss, Hearing aids

## Abstract

**Background:**

If sufficient speech discrimination is no longer achieved with conventional hearing systems, an audiological indication for a cochlear implant (CI) is given. However, there are no established target criteria for CI aftercare with regard to the level of speech comprehension to be achieved. The aim of this study is to validate an existing predictive model for speech comprehension after CI provision. This is applied to different patient groups.

**Materials and methods:**

The prospective study included 124 postlingually deaf adults. The model is based on preoperative maximum monosyllabic recognition score, aided monosyllabic recognition score at 65 dB_SPL_, and age the time of implantation. The model was investigated with regard to prediction accuracy for monosyllabic recognition with CI after 6 months.

**Results:**

Mean speech discrimination improved from 10% with hearing aid to 65% with CI after 6 months, with a statistically significant improvement in 93% of cases. Deterioration of aided unilateral speech discrimination was not observed. The mean prediction error was 11.5 percentage points in the cases with preoperative scores better than zero and 23.2 percentage points in all other cases.

**Conclusion:**

Cochlear implantation should also be considered in patients with moderately severe to severe hearing loss and insufficient speech discrimination with hearing aids. The model based on preoperatively measured data for predicting speech discrimination with CI can be used in preoperative consultation and in the context of postoperative quality assurance.

## CI indication

The main objective of the treatment of hearing-impaired patients is to restore or improve speech perception. The provision of sound-amplifying hearing aids is initially the therapy method of choice. Only when these or other hearing systems can no longer achieve sufficient speech perception do the audiological prerequisites exist for the indication of cochlear implant (CI) provision. According to the German S2k guideline “Cochlea-Implantat Versorgung” (Cochlear Implant Care), this can be considered in the case of a monosyllabic score with optimized hearing aid fitting of up to 60% at a presentation level of 65 dB_SPL_ [1]. Thus, even in people with comparatively good pure-tone audiogram, cochlear implantation may be indicated if sufficient speech perception is not achieved with hearing aids.

## Quantification of speech perception

Various tests are available for quantifying speech perception. The Freiburg monosyllabic test is an established standard procedure in clinical hearing aid and CI diagnostics as well as in scientific studies [1, 9, 10, 18, 22, 23]. In hearing aid fitting, the maximum achievable word (monosyllabic) recognition score (WRS_max_) gives an indication of the speech perception for colloquial speech to be aimed for with hearing aids and can be used as a target criterion for hearing aid fitting [12, 17, 25]. However, there are no comparable established target criteria for CI aftercare and fitting. One reason for this is the heterogeneity of the patient groups, the insufficient determinability of the functional integrity of central nervous processing, the insufficient knowledge of corresponding influencing factors, and the difficulty of controlling these factors in large clinical studies [3, 4, 8, 16, 20]. The preoperative estimation of the speech perception that can be expected with a CI system is particularly important for people who still have speech perception.

In an earlier study [13] it was shown that the preoperatively measured WRS_max_ can be used as a lower estimator for the word recognition scores achievable with CI, WRS_65_ (CI). More recent studies confirm this result [14, 27]. Recently, for patients with hearing loss of < 80 dB_HL_, a prediction model for the WRS_65_ (CI) to be expected with CI has been developed, based on the preoperatively known variables of WRS_max_, word recognition scores with hearing aid at 65 dB_SPL_, WRS_65_ (HA), and age at the time of surgery [14], see Eq. 1.

1 $$\mathrm{WRS}_{65}\left(CI\right)\left[{\%}\right]=\frac{100}{1+e^{-\left(\beta _{0}+\beta _{1}\cdot \mathrm{WRS}_{\max }+\beta _{2}\cdot age+\beta _{3}\cdot \mathrm{WRS}_{65}\left(HA\right)\right)}}$$

with

β_0_ =: 0.84 ± 0.18
β_1_ =: 0.012 ± 0.0015 1/%
β_2_ =: −0.0094 ± 0.0025 1/year
β_3_ =: 0.0059 ± 0.0026 1/%

From the signs of the parameters it can be seen that a higher age has a negative effect on WRS_65_ (CI), whereas a higher WRS_max_ or WRS_65_ (HA) leads to a higher speech perception with CI.

The prediction value determined with Eq. 1 is currently used at the University Hospital Erlangen as a parameter for quality assurance and for individual preoperative counseling of CI candidates. Especially in cases with still substantial residual hearing, an individual prognosis is desirable [19, 24, 28]. The aim of this study is to apply the model developed in an earlier, retrospective study [14] in the context of a prospective study. For this purpose, the application to two groups of patients with different preoperative WRS_max_ (equal to or greater than zero), independent of pure-tone hearing loss, was investigated with regard to the prediction error.

## Patients and method

The data presented were collected during routine clinical examinations of CI pre-diagnostics as well as basic and follow-up therapy of postoperative CI treatment. The prospective study was approved by the responsible ethics committee (AZ 60_20B) and registered with the German registry for clinical studies (DRKS00023351).

### Patient characteristics

In total, the data of all adult patients who were fitted with a Nucleus CI (Cochlear Ltd., Sydney, Australia) in the period October 2020 to December 2021 in the Ear, Nose and Throat Clinic, Head and Neck Surgery of the University Hospital Erlangen were evaluated in this study. Inclusion criteria were a postlingually developed hearing disorder, German as native language, CI indication according to the current German CI guidelines [1] due to sensorineural or mixed hearing loss, and at least 6 months of rehabilitation in our CI center. Exclusion criterion was a cognitive impairment that would have influenced the performance of the speech audiometry. Patients with an existing ipsilateral CI fitting (reimplantation) were also excluded. Currently, preoperative data as well as postoperative word recognition scores for a period of at least 6 months after surgery are available for 124 patients. The patient population consisted of 73 men and 51 women. The mean age at the time of CI surgery was 65.0 ± 13.9 years. All patients were using a hearing aid on the later implanted side at the time of CI pre-diagnosis. The hearing loss for air conduction was determined as the mean value over the four octave frequencies 0.5, 1, 2, and 4 kHz (4FPTA). For hearing thresholds beyond the maximum possible presentation levels of the audiometers, a value of 130 dB_HL_ was imputed. This resulted in a mean hearing loss of 92 ± 21 dB_HL_. The majority of cases were unilateral CI provision with a mean pure-tone hearing loss on the contralateral side of 54 ± 26 dB_HL_. In 21 cases, the contralateral side was already provided with a CI. The speech processor used by 100 CI recipients was a behind-the-ear processor (CP1000), 24 patients wore an off-the-ear processor. The demographic details are summarized in Table 1.

**Table 1:** Demographics of the patient population

Demographics								
**Gender**	*Male*			*Female*				
*N* =	73			51				
–	*Minimum*			*Maximum*		*Median*		
**Age (years)**	25			86		66		
**Duration of deafness (years)**	0			79		20		
**Duration of hearing aid use (years)**	0			60		10		
**Hearing loss ipsilateral (dB**_**HL**_**)**	47			130		87		
**Hearing loss contralateral (dB**_**HL**_**)**	3			95		63		
**WRS**_**max**_** ipsilateral (%)**	0			100		23		
**WRS**_**65**_**(HA) (%)**	0			50		0		
**Etiology:**	*Unknown*	*Infection*	*Menière*	*Trauma*	*Cholesteatoma*	*Ototoxic drugs*	*Meningitis*	*Syndromes*
*N* =	84	13	8	8	3	3	2	3
**Implant type**	*CI612*			*CI632*		*CI622*		
	32			91		1		
**Processor type**	*CP1000*			*Kanso1*		*Kanso2*		
	100			21		3		
**No. of therapy sessions**	*Minimum*			*Maximum*		*Median*		
	6			12		9.5		

### Measurements

Pure-tone measurements (air conduction) and speech audiometric measurements (Freiburg monosyllabic test, DIN 45621) were analyzed. Of the preoperative measurements, the 4FPTA, the maximum word (monosyllabic) recognition score in the speech audiogram according to DIN 45621 (WRS_max_ [%]), and the monaural word recognition score measured with hearing aid in the free field at 65 dB_SPL_ (WRS_65_ (HA) [%]) were used. The hearing aids were technically checked in advance. In particular, in situ measurements were taken to ensure that the settings resulted in the adequate prescription target [5]. Of the postoperative measurements, the word recognition score with CI in the free field at 65 dB_SPL_ (WRS_65_ (CI) [%]) was evaluated.

The measurements in the free field were performed in a soundproof cabin (6 × 6 m). The loudspeaker was placed 1.5 m in front of the patient (0°azimuth). The contralateral ear was properly masked with broadband noise via headphones, if necessary.

### Data analysis

The analysis and the creation of the figures were carried out using the software package Matlab® R2019b (MathWorks, Natick/MA, USA). Three preoperative variables—WRS_max_, WRS_65_ (HA) and age (see Eq. 1)—were used to predict WRS_65_ (CI). The prediction error was quantified using the median absolute error (MAE).

## Results

### Preoperative audiometry

**Fig. 1:**
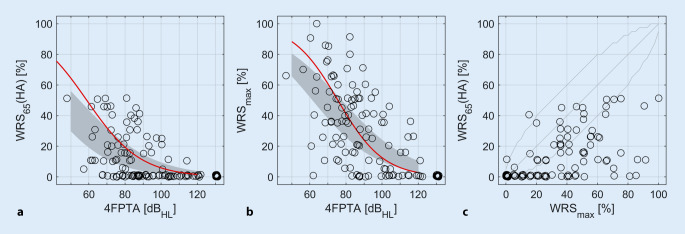
Relation of the preoperative variables mean hearing threshold, *4FPTA*, word recognition score with hearing aid, *WRS*_*65*_* (HA)*, and maximum word recognition score, *WRS*_*max*_. **a** The WRS_65_ (HA), free field at 65 dB_SPL_, as a function of the 4FPTA. **b** The relation between 4FPTA and WRS_max_. **a,** **b** The *red lines* represent the mean WRS_65_ (HA) and WRS_max_ from a previous study [11]. The *gray areas* correspond to the confidence interval for the mean value of the current data. **c** The WRS_65_ (HA) as a function of WRS_max_ together with the upper and lower critical differences from [10]

Figure 1 describes the relationships between the 4FPTA, the maximum word recognition score, and the word recognition score measured in the free field at 65 dB_SPL_ with hearing aid. The red lines (Fig. 1a, b) represent the mean WRS_65_ (HA) and the WRS_max_, respectively, as a function of the 4FPTA from a previous study [11] in a population of hearing aid users. In all cases, the WRS_65_ (HA) was less than or equal to 50% and thus well within the indication range for CI provision [1]. The WRS_max_ was above 50% in about one fifth of the cases (*n* = 23; Fig. 1c).

### Postoperative audiometry

**Fig. 2:**
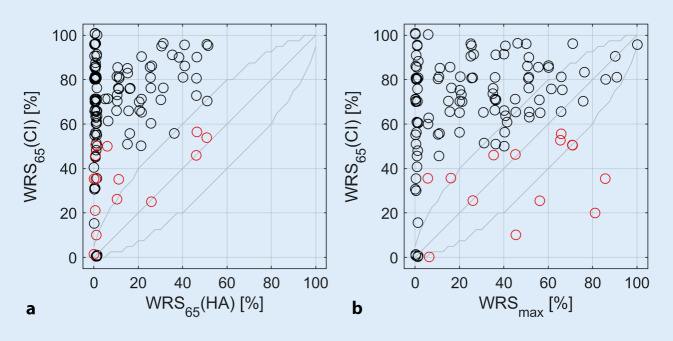
Word recognition scores with cochlear implant (*CI*) measured postoperatively after 6 months, *WRS*_*65*_* (CI)*, compared to preoperative measurements of word recognition. **a** WRS_65_ (CI) versus WRS_65_ (HA), free field at 65 dB_SPL_. **b** WRS_65_ (CI) versus the WRS_max_. The critical cases discussed in Fig. 4 are shown *in red*

Figure 2 shows the word recognition scores with CI measured after 6 months of hearing experience with a CI, depending on the preoperative WRS_65_ (HA; Fig. 2a) and WRS_max_ (Fig. 2b), respectively. Mean scores improved from 10% with HA to 65% with CI after 6 months. In 90% of cases (*n* = 112), scores improved by at least 20 percentage points (%-points). A statistically significant improvement of word recognition scores was observed in 93% of the cases (*n* = 115) after 6 months. The significance was tested using the critical differences according to Winkler and Holube [10]. No deterioration in speech perception was observed for any of the cases. In 116 cases the WRS_65_ (CI) was within the confidence interval of the Freiburg test or better than the preoperative WRS_max_. In eight cases, the WRS_65_ (CI) was significantly [10] lower than the WRS_max_.

**Fig. 3:**
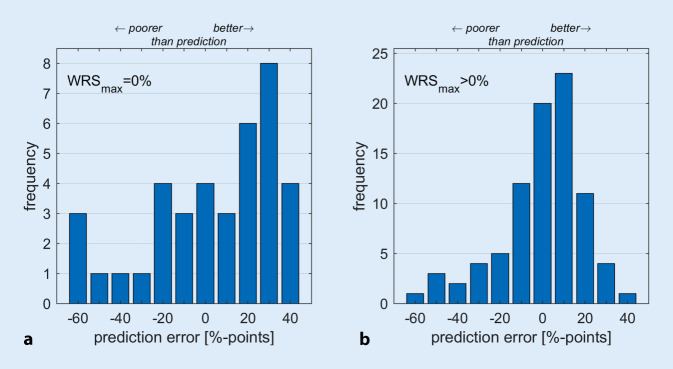
Difference between word recognition measured at 6 months postoperatively with cochlear implant (CI), *WRS*_*65*_* (CI)*, and the predicted word recognition for this time point for two subpopulations. **a** The 39 cases with preoperative WRS_max_ equal to 0, **b** the 85 cases with preoperative WRS_max_ better than 0

Figure 3 presents the differences between the WRS measured at 6 months postoperatively, WRS_65_ (CI), and the value predicted for this time point according to Eq. 1 for two subpopulations, WRS_max_ = 0% and WRS_max_ > 0%, of this study. This classification is motivated by a previous study [13]. Figure 3a summarizes the differences of the 39 patients with a preoperative WRS of 0. Positive values correspond to better-than-predicted scores. The median absolute error for prediction here is 23.2% points. There is no correlation between predicted and measured word scores (*p* > 0.23). Figure 3b shows the summary of the differences of the 85 patients with a preoperative WRS_max_ better than 0. The median absolute error for prediction here is 11.5% points. For 47 cases, the error was in a corridor of ± 10%-points, 32 cases fell short of the prediction by more than 10%-points, while for 45 cases, word recognition was observed to be more than 10%-points above the prediction.

**Fig. 4:**
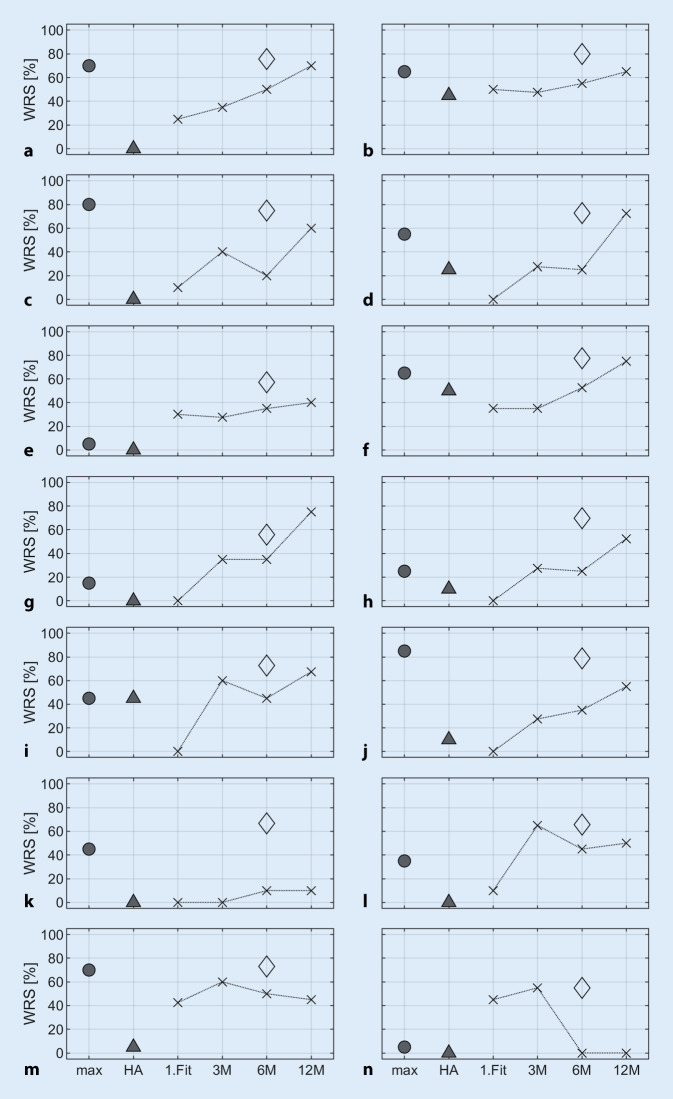
Time course of word recognition scores (*x*) with cochlear implant (CI) of all cases with unexpectedly poor speech perception with preoperative WRS_max_ better than 0 (**a–n**). The *circles* correspond to the preoperatively measured WRS_max_, the *triangles* represent the monosyllabic scores achieved with hearing aid. The *diamonds* correspond to the predicted scores with CI for 6 months postoperatively

Figure 4 describes selected individual cases from the subpopulation summarized in Fig. 3b over time. All cases from Fig. 3b that failed to achieve the predicted score by more than 20%- points are shown here. These are referred to below as cases with *unexpectedly poor speech perception. *Of these 14 cases, nine (Fig. 4a–i) achieved the predicted score within a window of 20%- points after 12 months. Another case (Fig. 4j) shows a slower increase in word recognition, which suggests a delayed achievement of the prognosis with progressing therapy. In the remaining four of 85 cases (4.7%) with a preoperative WRS_max_ better than zero, no improvement in speech perception is foreseeable due to a very flat slope (Fig. 4k) or moderately (Fig. 4l, m) to strongly (Fig. 4n) fluctuating speech perception.

## Discussion

In this prospective study, mean speech word recognition scores improved from 10% with HA to 65% with CI after 6 months. Here, a significant [10] improvement was observed for 93% of the cases. In none of the cases was there a worsening of speech perception. Furthermore, a model proposed in a previous retrospective study ([14], Eq. 1) was evaluated to predict monosyllabic scores after 6 months of CI rehabilitation. The prediction error was 11.5%-points in cases with preoperative residual monosyllabic scores (WRS_max_ > 0%) and 23.2%-points in those with WRS_max_ = 0%.

### Essential parameters

Initially, the model was developed using data from patients with preoperative hearing loss better than 80 dB_HL_. For the majority (92%), a WRS_max_ above zero was measured at that time. Therefore, it initially seems reasonable to limit the scope towards higher pure-tone hearing losses to cases with WRS_max_ > 0. For this group (*n* = 85), at least minimal functionality of the auditory nerve is given. Here, unexpectedly poor speech perception was observed in only four cases, even in the longer term, which suggests that the preoperatively predicted word recognition will not be achieved. By contrast, in the group with WRS_max_ equal to zero (*n* = 39), no such information about auditory nerve function is available. Therefore, the prediction error for this group is expectedly larger.

The WRS_max_ was introduced in a previous paper as a minimum predictor for the WRS_65_(CI) [13]. In the current patient population, the WRS_max_ is met or exceeded in 116 cases (93.5%). In eight cases (6.5%) the WRS_65_(CI) is significantly [10] lower than the WRS_max_. Overall, this shows a broad agreement with a study conducted elsewhere [27], which also points to the great importance of the WRS_max_ in the context of CI provision.

In a study by Shafieibavani et al. [26], different modeling approaches were compared with each other. The authors report mean prediction errors of 20%- to 22%-points. This analysis was based on 2489 cases treated between 2003 and 2018 in either a German, a US, or an Australian institution. The published preoperative audiometric findings suggest that a large proportion of these were patients who had a WRS_max_ close to zero. The model errors found there are of the same order of magnitude as the prediction errors shown in Fig. 3a, with an MAE of 23.2%-points for the group of patients with WRS_max_ equal to zero.

The MAE of 11.5%-points found for the population with a WRS_max_ better than zero justifies post hoc the application of the predictive model described here [14]: Limiting the population to functional residual hearing in the sense of still measurable speech perception contributes significantly to the reduction of the prediction error. In the previous study [14], the inclusion criterion was limited to cases with a hearing loss of 80 dB_HL_ or less. In this group, the WRS_max_ is usually greater than zero [15]. For candidates with a hearing loss greater than 80 dB_HL_, a WRS_max_ better than zero was observed for 44 of 82 cases (54%). These cases are included in Fig. 4b. In summary, therefore, the modification of the original scope of the prediction model for hearing losses of 80 dB_HL_ or less to word recognition of WRS_max_ > 0 is justified [14], as the MAE remains almost unchanged. Application to cases with WRS_max_ = 0 is possible but results in a larger prediction error (see Fig. 3a). Via Eq. 1, the predicted monosyllabic score for these cases is determined by the constant β_0_ and the age. The constant β_0_ represents the mean word recognition with CI without the individual correction influences of the other three variables.

### Quality assurance in CI therapy

The predictive value presented here can be used together with the WRS_max_ as a quality assurance parameter. This results in a corridor within which the postoperative word recognition score with CI should be. The deviation from the predicted value in combination with the deviation from the lower expected value, the WRS_max_ [13], enables early identification of cases with *unexpectedly poor speech perception* (Fig. 4), and the initiation of appropriate additional measures in the context of basic and follow-up therapy. First, pathophysiological causes and technical malfunctions [2] must be ruled out. Then, additional technical processor adjustments or modification and intensification of hearing and speech therapies, but also a review of user behavior and appropriate counseling [6, 7, 21] should be considered. In the follow-up of the cases presented here, such quality assurance was performed and after 3 months of CI experience, the WRS_65_(CI) was compared with the preoperative WRS_max_ and the predicted value according to Eq. 1. In an interdisciplinary case review, complementary therapy modifications were then initiated if necessary. This may have resulted in the actual performance being slightly above the prediction (see Fig. 3).

The model presented here is associated with a low prediction error for cases with WRS_max_ > 0. In our population, this applies to about two thirds of all postlingually deafened adult CI candidates. This group in particular has understandable reservations about undergoing an operation necessary for CI provision. In this respect, the assessment of the success of the therapy is particularly useful for these CI candidates.

Although the prediction model was developed using data from patients with CI systems from one manufacturer, the dependencies found should also be applicable to provisions with other systems. To determine the quantitative dependencies for different CI systems and rehabilitation concepts, further studies at other institutions would be desirable. In principle, it is desirable that the prognosis of speech perception with CI is based on preoperative data. In addition to the values presented here, results of future hearing diagnostics [20] could also contribute to the model. Furthermore, intraoperative measurements could also be used for this purpose and thus serve quality assurance. Further studies are necessary for this.

## Practical conclusion

- The provision of cochlear implant (CI) patients with an average hearing loss in the order of 60 dB_HL_ and insufficient speech perception with hearing aids is a therapy option.
- The model for predicting word recognition with CI based on preoperatively measured data can be used in counseling CI candidates and for quality assurance in postoperative rehabilitation. The limitation to a population with preoperative monosyllabic scores better than zero reduces the prediction error.
- The model enables early identification of cases with unexpectedly poor speech perception.

## Declarations

For this article no studies with human participants or animals were performed by any of the authors. All studies mentioned were in accordance with the ethical standards indicated in each case.

The supplement containing this article is not sponsored by industry.
