# Erratum to: Validation of a predictive model for speech discrimination after cochlear impIant provision

**DOI:** 10.1007/s00106-023-01332-8

**Published:** 2023-07-17

**Authors:** Ulrich Hoppe, Anne Hast, Thomas Hocke

**Affiliations:** 1 Audiologische Abteilung, Hals-Nasen-Ohrenklinik, Kopf- und Halschirurgie, Universitätsklinikum Erlangen, Waldstr. 1, 91054 Erlangen, Germany; 2 Cochlear Deutschland GmbH & Co. KG, Mailänder Str. 4a, 30539 Hannover, Germany

**Erratum to:**

**HNO 2023**

10.1007/s00106-023-01285-y

The article *Validation of a predictive model for speech discrimination after cochlear impIant provision*, written by Ulrich Hoppe et al., was originally published electronically on the publisher’s internet portal on 05.04.2023 without open access. With the author(s)’ decision to opt for Open Choice the copyright of the article changed to © The Author(s) 2023 and the article is forthwith distributed under a Creative Commons Attribution 4.0 International License, which permits use, sharing, adaptation, distribution and reproduction in any medium or format, as long as you give appropriate credit to the original author(s) and the source, provide a link to the Creative Commons licence, and indicate if changes were made.

The images or other third party material in this article are included in the article’s Creative Commons licence, unless indicated otherwise in a credit line to the material. If material is not included in the article’s Creative Commons licence and your intended use is not permitted by statutory regulation or exceeds the permitted use, you will need to obtain permission directly from the copyright holder. To view a copy of this licence, visit http://creativecommons.org/licenses/by/4.0/.

